# The Malignant Gastrointestinal Neuroectodermal Tumor (GNET): A Distinct Entity and the Challenging Differential Diagnosis with Mesenchymal, Lymphoid, and Melanic Tumors: A Case Report and Brief Review of the Literature

**DOI:** 10.3390/diagnostics13061131

**Published:** 2023-03-16

**Authors:** Mădălina Boșoteanu, Miruna Cristian, Mariana Așchie, Radu Andrei Baz, Alina Marta Zielonka, Georgeta Camelia Cozaru, Luana Andreea Boșoteanu

**Affiliations:** 1 Faculty of Medicine, “Ovidius” University of Constanta, 900470 Constanta, Romania; 2 Department of Clinical Pathology, “Sf. Apostol Andrei” Emergency County Hospital, 900591 Constanta, Romania; 3 Department of Pathology, “Ovidius” Clinical Hospital, 905900 Constanta, Romania; 4 Center for Research and Development of the Morphological and Genetic Studies of Malignant Pathology—CEDMOG, “Ovidius” University of Constanta, 900591 Constanta, Romania; 5 Institute of Doctoral Studies, School of Medicine, “Ovidius” University of Constanta, 900573 Constanta, Romania; 6 Academy of Medical Sciences, 030167 Bucharest, Romania; 7 Department of Radiology, “Sf. Apostol Andrei” Emergency County Hospital, 900591 Constanta, Romania; 8 Clinica Sante, 060754 Bucharest, Romania; 9 Dermatology Department, “Elias” Emergency University Hospital, 011461 Bucharest, Romania

**Keywords:** diagnosis differential, gastrointestinal neoplasms, neuroectodermal tumors, sarcoma, clear cell

## Abstract

(1) Background: A malignant gastrointestinal neuroectodermal tumor (GNET) is an ultra-rare primary neoplasm with a distinctive histopathological, immunohistochemical, molecular, and ultramicroscopic profile, synonymous terminology with clear cell sarcoma-like tumor of the gastrointestinal tract. This case report aims to describe a case of GNET with challenging mesenchymal, lymphoid, and melanic tumor differential diagnosis. (2) Case presentation: We discuss the case of a 67-year-old male patient who presented with diffuse abdominal pain, intermittent lack of intestinal transit, and frequent episodes of nausea, followed by segmental resection of the jejunum and sigmoid colon. The patient had no relevant medical history. The surgical specimen underwent immunohistochemical staining and morphological evaluation. (3) Results: Histopathological analysis reveals a moderately homogeneous polyhedral-epithelioid and spindle cell neoplastic proliferation with a zonal discohesive pattern and extensive and focal fasciculated architecture. Twenty monoclonal antibodies were used for immunostaining, which allowed GNET to be diagnosed on the basis of the tumoral immune profile, characterized by positive reactivity of S100, SOX10, and CD 56. (4) Conclusions: The poor prognosis of GNET is highlighted in the present study, along with the vital importance of differential diagnosis issues with mesenchymal, lymphoid, and melanic tumors, which make the diagnosis difficult for both pathologists and clinicians.

## 1. Introduction

A malignant gastrointestinal neuroectodermal tumor (GNET) is an ultra-rare primary neoplasm with a distinctive histopathological, immunohistochemical, molecular, and ultramicroscopic profile, synonymous terminology with a clear cell sarcoma-like tumor of the gastrointestinal tract (CCS-like tumor of the GI tract) [1].

Although it shares common molecular genetic abnormalities with the condition known as malignant gastrointestinal neuroectodermal tumor (GNET), clear cell sarcoma is a rare sarcoma that infrequently develops as a primary tumor in the gastrointestinal tract. However, it is distinguished from GNET by its morphological and immunohistochemical findings [1]. Since there are so few occurrences of this tumor described in the literature, more information is needed about its behavior and diagnosis. GNET can mimic several different tumors, making it difficult for pathologists and physicians to diagnose.

Currently, a CCS-like tumor of the gastrointestinal tract (malignant gastrointestinal neuroectodermal tumor) most likely represents a distinct entity from clear cell sarcoma (CCS) and is discussed in the 5th Edition of WHO Digestive system tumors volume. Many experts favor the name “CCS” when Melan-A, HMB45, or MITF are expressed and the term “malignant GNET” when these markers are not present [1,2,3,4,5].

In contrast to tumors described as gastrointestinal (GI) tract CCS, which have a higher median age (57 years; range: 35–85 years) and a more significant percentage of male patients (85%), the incidence of tumors reported as GNET has a median patient age of 33 years (range: 10–81 years) and an even sex distribution [1].

Rosai [6], stating prior studies [7,8], first summarized the descriptive evolution of this entity. Rosai [6] highlighted the development of a new tumor of the digestive tract that shares cytogenetic characteristics with clear cell sarcoma of tendons and aponeuroses (CCSTA), namely *t*(*12*;*22*)(*q13*;*q12*) chromosomal translocation and the emergence of the *EWS-ATF1* fusion transcript [9].

As the hypothesis progressed, Stockman et al. [10] established this specific type of tumor as “GNET”, validating the *EWSR1* gene rearrangement using molecular biology techniques and the absence of melanogenesis using ultrastructural data. To date, at this moment, 111 cases have thus far been documented in the literature [10,11]. GNET’s histological characteristics can be partially superimposed with other types of gastrointestinal tract lesions. As a result, using ancillary techniques is necessary to finish the diagnosis.

This study will report a case of GNET with intestinal occlusion as a clinical symptom of the jejunal tumor’s colonic development. Mesenchymal, lymphoid, and melanic tumors present differential diagnosis challenges due to the neoplasm’s microscopic characteristics.

These issues were solved by using immunohistochemistry, which is crucial for finalizing the diagnosis. Moreover, having as a starting point the presentation of this case, a literature review was carried out, oriented toward the clinical, cytogenetic, and electron microscopy traits of GNET.

## 2. Case Presentation

### Clinical Findings

**Figure 1:**
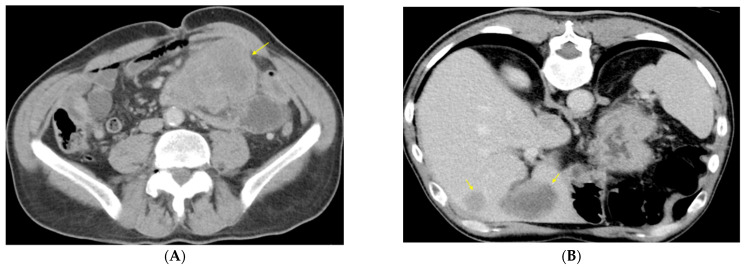
Abdominal CT scan at the first diagnosis, with i.v. administration of contrast material (venous phase shown): (**A**) large heterogeneous, hypoenhancing mass with infiltrative growth located in the left flank near jejunal loops and descending colon; (**B**) liver nodules/metastases hypoattenuating on unenhanced CT (not shown), enhancing at their periphery but less than surrounding liver parenchyma following contrast administration (A-yellow arrows).

A 67-year-old male patient without significant history lately showed diffuse abdominal pain accompanied by the intermittent absence of intestinal transit, repeated episodes of nausea and vomiting, and symptoms that progressively worsened; for these reasons, he was hospitalized for diagnosis and therapy. Physical examination revealed a moderately distended, diffusely painful abdomen, with palpation of a voluminous, painful mass in the left abdomen. Among the laboratory tests significant for diagnosis, leukocytosis of 17.88/mm^3^ was noted. The computed tomography (CT) of the abdomen and pelvis revealed a large heterogeneous, hypoenhancing mass in the left flank near the jejunal loops, measuring 12 × 8.5 × 14.5 cm (Figure 1A). The described mass exhibited intensely inhomogeneous iodophilia due to necrotic areas. The liver presented nodular lesions (10) in multiple segments (Figure 1B) without invading the portal vein or the hepatic pedicle. 

The lesions were hypodense, hypoattenuating, and randomly distributed, with necrotic center and perilesional perfusion disorders, with a maximum diameter of 6.4 × 4.7 cm at the level of the VIth segment—CT aspect compatible with liver metastases. No extrahepatic metastatic disease was found on the CT scan. Subdiaphragmatic lymphadenopathy with tumoral features was also noted. Colonoscopy performed up to the level of the sigmoid colon would have had no diagnostic value due to the impossibility of continuing the exploration because of the abovementioned tumor mass. Surgery was performed after considering the symptoms, and CT imaging was also performed. The patient underwent an exploratory laparotomy with segmental resection of the jejunum and sigmoid colon, entero-enteral isoperistaltic mechanical end-to-end anastomosis, and left temporary terminal colostomy. The surgical diagnosis was as follows: mechanical intestinal occlusion by an abscessed voluminous perforated tumor of jejunum with invasion in the sigmoid colon, generalized acute sero-purulent peritonitis, jejunal mesenteric lymphadenopathy, multiple liver metastases, and secondary anemia. 

The specimens obtained from the surgical intervention were sent to the Pathology Department for histopathological examination. 

The immediate postoperative evolution was good with the resumption of the transit for feces and gases at the level of colostomy. Still, the patient’s condition gradually deteriorated six weeks after the surgical intervention, with the patient presenting uncontrolled algic syndrome, inappetence, and jaundice. The laboratory tests showed cytolysis syndrome, cholestasis, azotic retention syndrome, hypoalbuminemia, and hyponatremia. The follow-up treatment consisted of a pain reliever with minor opioids, symptomatic (endovenous infusions of hydration and hydro electrolytic rebalancing), and palliative; but unfortunately, the patient died. 

## 3. Results

### 3.1. Histopathological Examination

The surgical specimen consisting of a 25 cm small intestine and an 18 cm large intestine, which strongly adhere to each other, reveals in the jejunal segment a 13 cm-length ulcer-infiltrative lesion with circumferential growth. The section surface shows a white-grayish translucent aspect with transmural involvement and extension to the mesentery and sigmoid colon. The consistency is variable due to the alternate of the firm and friable areas. The enteral wall corresponding to the described lesion highlights a 5 × 4 cm continuity solution with gray-yellowish deposits. Pericolonic adipose tissue exhibits vascular thromboses associated with 0.5–0.8 cm whitish nodules. 

**Figure 2:**
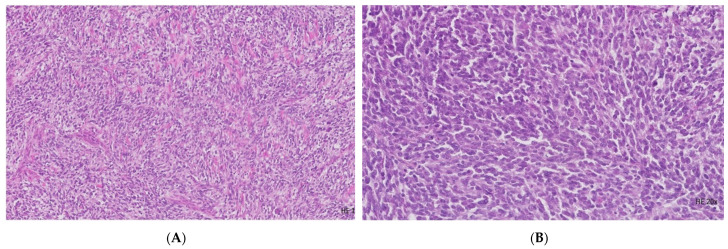
Histopathological evaluation. (**A**) The image shows a relatively uniform medium-sized polyhedral-epithelioid and spindle cell neoplastic proliferation with a zonal discohesive pattern, massive and focal fasciculated architecture (H&E, 10×); (**B**) The image reveals moderate cito-nuclear pleomorphism, with coarse chromatin and conspicuous nucleoli with high mitotic activity (H&E, 20×).

The surgical specimen was processed in the Pathology Department using standardized analytically validated protocols: fixation in 10% formalin, paraffin embedding, sectioning at 4-μm, and staining with hematoxylin and eosin (H E). Microscopically, a relatively discohesive pattern and massive and focal fasciculated architecture are observed (Figure 2A). The malignant population is confined predominantly to the submucosa and exhibits a dissecting effect on the muscular and serosal layer of the jejunal wall, with extrinsic colonic invasion, up to the mucosa level. The cellular features comprise moderate cito-nuclear pleomorphism, coarse chromatin, and conspicuous nucleoli (Figure 2B). Scattered multinucleated osteoclast-like giant cells are evident. The mitotic activity is high (30 mitoses/10 high-power fields HPFs) with atypical mitotic figures. Areas of necrosis are obviously apparent in approximately 50% of the tumoral volume. Stroma exposes broad areas of fibrohyalinization. Lymphovascular invasion (LVI) with the presence of neoplastic emboli and perineural infiltration (PNI) is identifiable. Two of the 20 lymph nodes sampled from the mesenteric and pericolic adipose tissue display the existence of metastases of 7 mm in maximal diameter.

### 3.2. Immunohistochemical Evaluation

An immunohistochemical evaluation was performed using several monoclonal antibodies (Table 1). The applied immunohistochemical techniques complied with the manufacturer’s recommendations (Ventana Medical Systems/Roche Tissue Diagnostics and Cell Marque Tissue Diagnostics).

**Figure 3:**
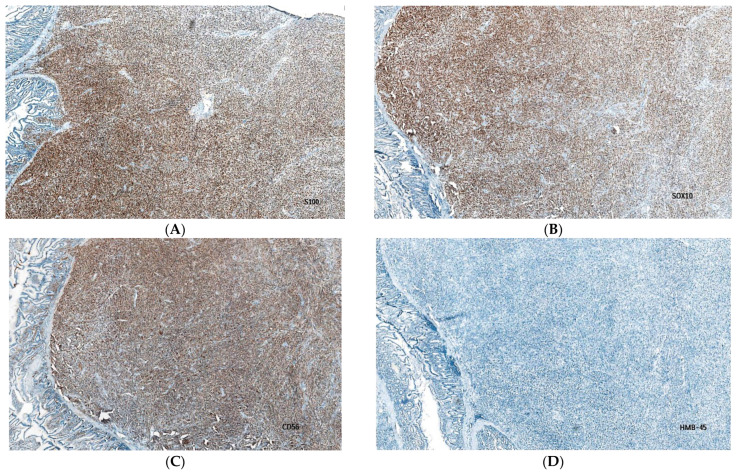

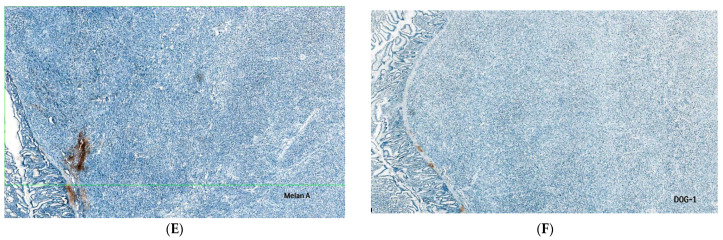
Immunohistochemical evaluation of the surgical specimen. (**A**) The image shows a 95% positive immunostain for the S100+ biomarker (IHC; 40×). (**B**) The SOX10 biomarker was positive in 90% of the cells within the tumor (IHC; 40×). (**C**) The image shows positive cells for the CD56 biomarker. (**D**) Negative immunostain for HMB-45 biomarker (IHC; 40×). (**E**) Negative immunostain for Melan-A biomarker (IHC; 40×). (**F**) The image shows tumor cells negative for the DOG-1 biomarker (IHC; 40×).

The tumoral immune profile (Table 2) is characterized by a positive reaction for S100 protein (Figure 3A), SRY-related HMG-box 10 (SOX10) protein (Figure 3B), cluster designation (CD) 56 (Figure 3C), and negative immunoexpression for human melanoma black HMB45 (Figure 3D), melanoma antigen (Melan-A), (Figure 3E), microphthalmia transcription factor (MITF), tyrosinase, desmin, discovered on GIST 1 (DOG-1) (Figure 3F), c-kit protooncogene, CD34, CD57, leukocyte common antigen (LCA), pan-cytokeratin AE1/AE3, epithelial membrane antigen (EMA), neurofilament (NF), synaptophysin (SYN), and chromogranin A (CG-A). The multiplication rate of the malignant cells was significant, consisting of a 40% index of proliferation cell marker Ki-67. 

### 3.3. Final Diagnosis

Based on the morphology and tumoral immunoprofile, a diagnosis of malignant gastrointestinal neuroectodermal tumor (GNET) was made.

## 4. Discussion

Kandler T. et al. claim that although the median age at diagnosis ranges from 33 to 36 years [1,7,10,11,12,13], GNET patients exhibit a wide range of age distribution and show no predilection for either gender.

In other investigations by Chang B. et al., Zambrano E. et al., Stockman D.L. et al., and Damle A. et al., patients typically exhibit intestinal symptoms such as abdominal discomfort, distension, obstruction, ascites, pelvic effusions, or abdominal tumors, either clinically or on imaging [7,10,12,13]. According to reports cited by Chang B. et al., Stockman D.L. et al., and Alyousef M.J. et al., nonspecific symptoms like anorexia, anemia, weight loss, high-grade fever, and weakness have been mentioned [4,10,12].

Contrarily, the Chang B. et al. analysis found that in 29% of instances, metastatic disease is frequently present at diagnosis [12]. This variety in clinical presentation is consistent with our patient, who presented with concurrent metastatic illness.

To date, there is still no agreement on the best systemic chemotherapy and targeted therapeutic alternatives for individuals who are not candidates for surgical excision because of significant metastases at initial presentation [14].

The problem in this case report is that it is hard to tell GNET apart from other mesenchymal, lymphoid, and melanocytic tumors. When CCS-like tumor of the gastrointestinal tract manifests clinically or radiologically as masses in the gastric or intestinal wall that frequently cause intestinal obstruction, several differential diagnoses can be made, including adenocarcinoma, gastrointestinal stromal tumor (GIST), leiomyosarcoma, neuroendocrine tumors (including carcinoid tumors), and lymphoma [5].

The absence of a melanoma clinical history helps rule out metastatic melanoma as a primary differential diagnosis, although conclusive evidence of an EWSR1 gene rearrangement is required [2]. These sarcomas can be recognized from those that do not have EWSR1 gene rearrangements on the basis of their appearance and immunohistochemistry [2].

Through their 16-case series, Stockman et al., who promoted treating GNET as a separate tumor entity rather than a variety of CCS, coined the term “GNET” in 2012 [10]; (Table 3). Only 111 cases were reported as of December 2021, according to Kandler T. et al. [11]; (Table 3), making it challenging to use the limited clinical, prognosticative, tumor staging, pharmacological, and treatment data that was available.

Furthermore, due to its rarity and similarity to other tumors, GNET is frequently misdiagnosed and treated improperly.

Primitive epithelioid, oval, or spindle tumor cells and sizable cells that mimic osteoclasts are typical features of this type of tumor. Because of the diverse histology, notably significant epithelioid or spindle cell components, these tumors might be mistaken for a range of other diagnoses, including a poorly differentiated carcinoma, such as a sarcomatoid carcinoma, according to a study by Chang B. et al. [12]. 

The absence of melanin pigment in every instance reported distinguishes CCS-like tumor of the gastrointestinal tract from CCS and melanomas affecting the gastrointestinal system [10,15,16,17,18]. The absence of melanin pigmentation does not preclude the diagnosis of CCS or melanoma because these diseases might have amelanotic forms. However, Lyle et al., Fukuda et al., Covinsky et al., and Pauwels et al. imply in their study that in 6 of 7 cases of traditional CCS of the digestive system where there was an active search for pigmentation, the melanin was found [6,19,20,21,22].

Fascicles of largely uniform spindle cells morphologically represent the majority of most of GIST with minimal pleomorphism, occasionally having paranuclear vacuolations or palisading, and sometimes displaying epithelioid and pleomorphic variants as well as, very rarely, osteoclast-like large cells. Zambrano et al. highlighted that a CCS-like tumor of the gastrointestinal tract differs from CCSTA in that it may feature osteoclast-like giant cells (OLGC) and does not display the distinctive markers of melanocyte development [7].

It is still debatable whether GI tract CCS and malignant gastrointestinal neuroectodermal tumors should be considered different tumors or the same tumor with varying degrees of differentiation [1,2].

A thorough IHC panel that includes cytokeratins, S100, and SOX-10 can also be used to identify the tumor as a GNET, and unlike carcinomas, GNET is cytokeratins negative [12].

IHC features that identify GNET tumors include S100 and SOX-10 protein positivity and the absence of melanocytic-specific markers like HMB-45 and Melan-A.

GNET frequently shows positive immunohistochemical results for S100 and SOX10 but negative results for markers more closely related to melanocytes, such as HMB45, Melan-A, and MITF. In any case, CD56 and SYN positive is a distinguishing trait of GNET diagnosis [2].

Routine immunohistochemistry should make it easy to rule out CCS-like tumor of the gastrointestinal tract, devoid of the expression of DOG1, CD117, or CD34, which are expressed by more than 90% of GISTs. DOG1 and CD34 are expressed by the majority of GISTs with KIT negative [5,23].

In cases when Melan-A, HMB45, or MITF are expressed, many specialists prefer the word “CSS”, while in cases where these markers are absent, they prefer the term “malignant GNET” [1,3,4,5,15]. In the present study, we discussed a case report that lends support to all of the arguments mentioned above.

The strategies incorporated by Stockman et al. that GNET tumors may arise from a primitive cell with a neural line of differentiation and no melanocytic characteristics that are connected to the autonomic nervous system [10,16] also supports the theory proposed by Antonescu et al. that GNET arises from neuroectodermal precursor cells with lost differentiating potential.

Stockman et al. have suggested that CCS-like tumor of the gastrointestinal tract be referred to as a “malignant gastrointestinal neuroectodermal tumor” due to the ultrastructural and immunohistochemical characteristics that support neural/neuroectodermal differentiation [5,10].

A retrospective analysis by Stockman et al. [10] revealed that *EWSR1* rearrangements were found in 12 of 14 cases (86%). The distinctive *t*(*12*;*22*)(*q13*;*q12*) and *t*(*2*;*22*)(*q34*;*q12*) chromosomal rearrangements of the GNET culminate in the production of the chimeric fusion proteins *EWSR1-ATF1* and *EWSR1-CREB1*, respectively [11]. In contrast, *EWSR1* rearrangements were present in 93.3% of the Chang et al. study cases, and both investigations used fluorescence in situ hybridization (FISH) to identify these rearrangements [12].

It still needs to be determined if some GNET patients’ better clinical outcomes result from their slowly developing disease biology or from more aggressive treatments, such as recurrent aggressive surgical operations and systematic therapy regimens. The patient in this case study had a poor clinical course and a poorer prognosis following the surgical treatment. Otherwise, it is argued that GNET is a biologically diversified condition, equivalent to all other cancer forms, although relatively rare.

To conclude, we consider that more research is required to develop standardized staging and therapy options for malignant GNET because there is currently no established procedure for its management.

**Table 3:** Previously reported cases with references.

Source	Year	Number of Cases	Comment
Fukuda et al. [20]	2000	1	Case report of a CCS arising in the transverse colon
Pauwels et al. [22]	2002	1	Case report of a CSS with morphological features resembling malignant melanoma.
Zambrano et al. [7]	2003	6	First described as an idiosyncratic type of gastrointestinal neoplasm that shared certain features with clear cell sarcoma of soft parts (melanoma of soft parts)
Rosai J [6]	2005	1	First descriptive evolution of clear cell sarcoma and osteoclast-rich clear cell sarcoma-like tumor of the gastrointestinal tract
Covinsky et al. [21]	2005	20	A report of a subset of GI tumors diagnosed as malignant melanoma by routine histopathologic evaluation represents CCS.
Antonescu et al. [16]	2006	3	A report of a subset of CCS that occurs preferentially in the gastrointestinal tract and shows little or no melanocytic differentiation, with a t(12;22) translocation resulting in the fusion of EWS (EWSR1) with ATF1
Lyle et al. [19]	2008	7	Molecular evaluation of 7 cases of CSS previously diagnosed as malignant melanoma
Stockman et al. [10]	2012	16	First introduction of term “GNET”
Alyousef et al. [4]	2017	1	-
Chang et al. [12]	2020	19	-
Damle et al. [13]	2021	1	-
Kandler et al. [11]	2022	23	-

## 5. Conclusions

In this case report, we described a case of a 67-year-old male patient without significant history, presenting with the clinical appearance of intestinal occlusion, diagnosed with GNET based on the morphology and immunophenotype, with an unfavorable clinical outcome and a worse prognosis after surgical intervention. 

The current report highlights the importance of early diagnosis of GNET because of its variable prognosis and highlights the critical importance of differential diagnosis problems with mesenchymal, lymphoid, and melanic tumors, which make the diagnosis challenging for pathologists and clinicians alike.

In conclusion, to help physicians design individualized treatment plans, it is critical to understand the biology of this disease.

## Figures and Tables

**Table 1:** Antibodies used for immunohistochemical evaluation.

Antibody	Isotype	Clone	Controls	Manufacturer
S100	IgG2a	4C4.9	Melanoma	Ventana Roche
SOX10	IgG	SP267	Melanoma, Skin Melanocytes	Cell Marque
CD56	-	MRQ-42	Pancreatic Islet Cells, Pancreatic Endocrine Cells, Neuroblastoma	Cell Marque
CD99	IgG1	O13	Pancreatic Islet Cells, Sertoli Cells of the Testis	Ventana Roche
Confirm anti-Melanosome	IgG1/K	HMB45	Melanoma, Skin	Ventana Roche
Confirm anti-MART-1/Melan-A	IgG1	A103	Melanoma, Normal Skin	Ventana Roche
Confirm anti-MITF	IgG1	C5/D5	Melanoma	Ventana Roche
Confirm anti-Tyrosinase	IgG2a	T311	Lung, Melanoma	Ventana Roche
Confirm anti-Desmin	IgG1/K	DE-R-11	Intestine	Ventana Roche
DOG-1	Rabbit Ig	SP31	Gastrointestinal stromal tumor	Cell Marque
C-kit (CD117)	-	9.7	Gastrointestinal stromal tumor	Ventana Roche
Confirm anti-CD34	IgG1	QBEnd/10	Appendix, Placenta, Tonsil	Ventana Roche
CD57	IgM/K	NK-1	Lymph Node, Tonsil	Cell Marque
CD45 (LCA)	IgG1/K	2B11 & PD7/26	Lymph Node, Tonsil	Ventana Roche
anti-Pan Keratin (AE1/AE3)	IgG1	AE1/AE3/PCK26	Intestine, Liver	Ventana Roche
Confirm anti-EMA	IgG2a	E29	Normal Pancreas	Ventana Roche
Neurofilament NF	IgG1/K	2F11	Brain	Cell Marque
Synaptophysin	IgG1	MRQ-40	Pancreatic Islet Cells	Cell Marque
anti-Chromogranin A (CG-A)	IgG1/K	LK2H10	Pancreas	Ventana Roche
Ki-67	-	30-9	Lymph Node, Tonsil	Ventana Roche

**Table 2:** The results of immunohistochemistry evaluation.

Antibody	Immunohistochemistry Evaluation
S100	Diffuse positive cytoplasmic and nuclear reactions
SOX10	High diffuse positive nuclear reaction
CD56	Positive cytoplasmic and cell membrane reactions
CD99	Low positive cell membrane reaction
Confirm anti-Melanosome	Absent reaction
Confirm anti-MART-1/Melan-A	Absent reaction
Confirm anti-MITF	Absent reaction
Confirm anti-Tyrosinase	Absent reaction
Confirm anti-Desmin	Absent reaction (Positive control)
DOG-1	Absent reaction
C-kit (CD117)	Absent reaction (Positive control)
Confirm anti-CD34	Absent reaction (Positive control)
CD57	Absent reaction
CD45 (LCA)	Absent reaction (Positive control)
anti-Pan Keratin (AE1/AE3)	Absent reaction (Positive control)
Confirm anti-EMA	Absent reaction (Positive control)
Neurofilament NF	Absent reaction
Synaptophysin	Absent reaction (Positive control)
anti-Chromogranin A (CG-A)	Absent reaction (Positive control)
Ki-67	A high positive nuclear reaction in about 40% of malignant cells.

## Data Availability

The data generated in the present case report are included in the figures and/or tables of this article.

## Abbreviations

CCS	clear cell sarcoma
CCS-like tumour of GI tract	Clear cell sarcoma-like tumor of the gastrointestinal tract
CCSTA	clear cell sarcoma of tendons and aponeuroses
CG-A	chromogranin A
CD56	cluster designation 56
CT	computed tomography
GNET	Malignant gastrointestinal neuroectodermal tumor
GI	Gastrointestinal
EMA	epithelial membrane antigen
FISH	fluorescence in situ hybridization
HPFs	high power fields
HMB45	human melanoma black 45
IHC	Immunohistochemistry
LCA	leukocyte common antigen
LVI	Lymphovascular invasion
Melan A	melanoma antigen
MITF	microphthalmia transcription factor
NF	Neurofilament
OLGC	osteoclast-like giant cells
PNI	perineural infiltration
SYN	synaptophysin
SOX10—SRY	related HMG—box 10 protein
